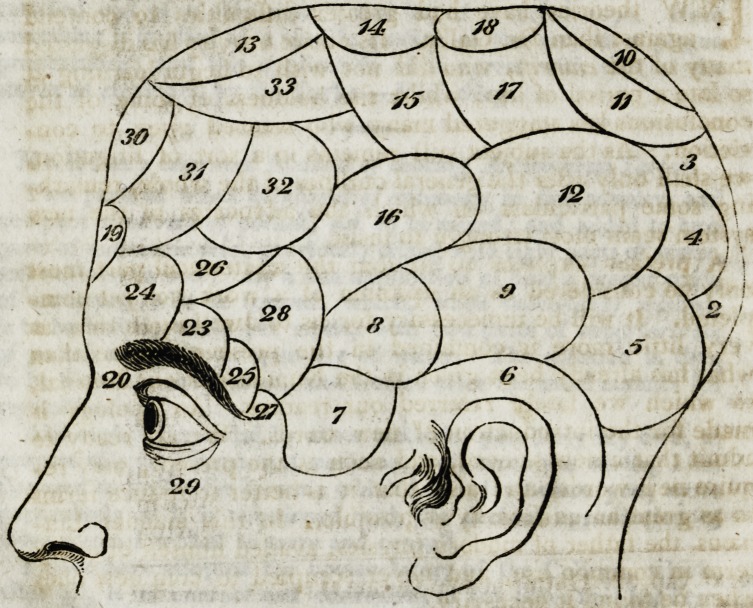# Book Reviews

**Published:** 1815-06

**Authors:** 


### CRITICAL ANALYSIS
### *OF RECENT PUBLICATIONS*
IN THE
### DIFFERENT BRANCHES OF PHYSIC, SURGERY, AND MEDICAL PHILOSOPHY.

*The Physiognomical System of Drs. Gall and Spurzheim; founded on an Anatomical and Physiological Examination of the Nervous System in general, and of the Brain in particular; and indicating the Dispositions and Manifestations of the Mind.* By J. G. SPURZHEIM, M.D.—Large 8vo. pp. 550, with plates. Baldwin and Co.

FEW theories have had greater difficulties to contend against than Dr. Gall's. Its entire novelty has disgusted many of the *emeriti*, who did not wish to begin learning at so late a period of life; whilst the boldness of some of the conclusions has staggered many who seemed open to conviction. As the subject still remains in a sort of litigation, we shall only offer the general outlines of the whole, remarking some particulars on which the advocates of the new system seem most strongly to insist.

A preface explains to us that the anatomical part must only be considered as an epitome of a work not yet completed. It will be unnecessary for us to dwell upon this, as very little more is contained in the present volume than what has already been given in the Numbers of our Journal, to which we lately referred our readers. An apology is made for the introduction of new words. We are ready to admit that a new science, and such is the present, may require new words, and also that it is better to reduce terms to as great an uniformity as possible. In this manner Linnæus, the father of modern physics, availed himself of every term in common use, and never scrupled to coin new ones when he found it necessary.

"The English language (says the preface) presents very few single words which express my conceptions of the peculiar faculties ties of the mind. Hence I was obliged either to speak by circumlocution, or to make new names. Now I think with Locke, that in this respect, we have the same right as our predecessors, and I therefore propose new single names, which I have formed, as much as possible, conformably to the spirit of the language. Having established different propensities as peculiar faculties of the mind, in order to designate propensity, I have taken the termination IVE as indicating the quality of producing, and NESS as indicating the abstract state; I have, therefore, joined IVENESS to different roots, among which I have given the preference to English words generally admitted. When I could not find any such, I chose Latin participles, which, in English, are so common even in expressions which denote a meaning similar to that which I look for, as destructiveness, productiveness, &c.

"I have further to make a few particular observations. In the nomenclature of the PROPENSITIES, I dislike the name physical love, because this propensity is neither more nor less physical than attachment, or any other inclination common to man and animals; and I could not admit some other expressions in order to denote this propensity, because I want names which indicate the faculty, and not at all any determinate action, whether in its use or its abuse. I have, therefore, adopted amativeness, like destructiveness.—It was difficult to make a name for the second organ, because there is no single word which indicates the love of offspring. Hence I took two Greek roots. I am aware that the name is long, but I could not say philogenitiveness, because the name ought to indicate love of producing offspring. As, however, progeny means offspring, and philoprogeny love of offspring, and philoprogenitiveness the faculty of producing love of offspring, I have adopted that term.—Inhabitiveness is composed of the English word inhabit, and the termination iveness.—It is true that adhesiveness is generally used merely in a physical sense, but was not this originally the case with many other words which bear now a mental signification? Attachment indicates only the effect of this faculty, and I require a name to express the faculty of producing such effect. It was naturally my desire to give the same termination to all the names which denote any propensity; and it seems to me that the sound attachiveness would be infinitely more disagreeable than adhesiveness, the signification of which only remains to be determined.—Combativeness is the propensity to combat.—Destructiveness is admitted in the language.—Constructiveness is, in our doctrine, the producing of construction.—I know that covetiveness is a pléonasm, but this fault is observed in many other words. Covet itself indicates propensity or wishing for; and I have added *iveness* solely for the sake of uniformity: otherwise I should have said covetingness.—Secretiveness is the propensity to secrete or conceal.

"The termination OUS indicates a SENTIMENT, as anxious, cautious, pious, conscientious, &c. and I should have been very glad of of finding similar adjectives for every primitive sentiment of the mind. When that has been the case, I have added NESS in order to express the abstract state, as cautiousness, righteousness, or conscientiousness.

"The names of the INTELLECTUAL FACULTIES are easily understood, and do not require any particular explanation.

"If, under any head of this nomenclature, there be any better name, or one which indicates more exactly any determinate faculty, but no determinate action or effect of the faculty, I shall be anxious to make use of it; for I am always disposed to acknowledge truth and every real improvement.

"As to style, my work, of course, presented, in the first instance, many Germanicisms and Gallicisms; and it probably still presents some of them. I am, however, indebted to my friend Alexander Walker, Esq. late Lecturer at Edinburgh, for bestowing upon its revisal such time as he could spare from other avocations."

On the modesty of the last sentence, we shall just stop to regret that, in correcting a foreign style, the author did not apply to an Englishman. It is not our business, who are not philologists, to ascertain which is the best language; but it is certain that the Scotch and the English are not the same. The Scotch grammar is, in all doubtful matters, influenced by the Latin; and there are some peculiarities in which it is stiffer, if we may be allowed the expression, than its guide. "*If* under any head there *be* any better name"—(see above). Now, there is no propriety in the use of this *be*, which is grating to an English ear, and the subjunctive mood would not have been necessary even in Latin.

On the addition of IVE to English words, and, where the author could not meet with them, his choice of Latin rather than Greek participles, it is pleasing to remark how exactly he has pursued Horne Tooke's etymologies, who called the adjectives ending in IVE "the potential active participles." As the Επεα Πτεροεντα are not in the hands of every medical reader, we shall transcribe the passage, to show how nearly Dr. Spurzheim has fallen into the opinion of our English etymologist, and his awkwardness wherever he has deviated from him.

"What is the termination of your *potential active adjective*?

"We have two terminations in English for this purpose, which is one more than enough; and yet our language has not hitherto availed itself of this useful abbreviation so extensively as it ought to have done. It is by no means familiar or in common use, as the potential passive adjective is; but is chiefly, though not intirely, confined to technical expressions.

"At the dawn of learning in this country, those who became acquainted with the Latin and French authors, perceived (and especially pecially when they came to translate them, or to repeat any thing after them) a convenient short method of expression in those languages, with which their own could not furnish them. Finding, therefore, this peculiarity, and not knowing whence it arose, as they proceeded, to be more familiar with those languages, they borrowed the whole Latin or French words in which the *abbreviation* they wanted was contained, instead of using their own periphrastic idiom, as formerly, or forming (as they should have done) a correspondent abbreviation in words of their own language. And thus, by incorporating those words, they obtained *partially* (for it extended no farther than the very words adopted) that sort of abbreviation to our language which it had not before.

"'That kind of composition which alters the terminations of words,' being nothing more than the addition of a word; and the addition which the Romans and Greeks made for this purpose being a word of their own language, whose *force* was consequently known to them, they could, upon occasion, add it to any verb they pleased, and its signification would be evident to all. For, though ισχυς and *vis*, by frequent use and repetition, were corrupted, and became in composition ικος and *ivus* in this *abbreviation;* yet the analogy which this termination would bear to the other words of the same sort, would justify the application of the same termination to any word where they might chuse to employ it. But that is not the case with us, for, as we have not obtained this abbreviation by 'that kind of composition which alters the terminations of words,' (i. e. by adding to one known word of our own, another known word of our own, expressive of the added circumstance) but only by adopting some of the *abbreviated* words themselves from other languages, we cannot so easily supply our defects and extend the advantage, unless we go on borrowing fresh abbreviated words, ready made to our hands, from the same sources.

"And this will appear plainly to any one who will please to examine our language, for we have not one single word of Anglo-Saxon origin, whose *potential mood active* is *adjectived.* Some attempts, indeed, have been made towards it, but without success; for Wilkins's '*vnwalkative*,' (for—one *who cannot walk*,) and other words of the same coinage, have never passed current amongst us. And it is well for the language that they have not, and that the greater part of these new-coined words have been rejected, because the persons who coined them being commonly affected, and always ignorant of the *force* of the *termination* they employed, would very greatly have injured and confounded the language by an improper application of the termination.

"All the *abbreviations* which we enjoy of this kind (i. e. the *potential active adjective*) are either borrowed from the Latin, and then they terminate in *ive*, as *purgative, vomitive, operative, &c.* or they are borrowed from the Greek, and then they terminate in *ic*, as *cathartic, emetic, energetic*, &c."

We

We have made this extract short in comparison of its importance, and of the pointed manner in which the subject is illustrated, in order that our readers in general, and Dr. S. in particular, should a new edition be called for, may see the advantages deriveable from an etymological knowledge of the terms employed. In the present instance, the professor has very high authority for *availing himself of this useful abbreviation.* But, when he says he has given a preference to our English words in general, or to Latin participles, he will now observe that, happily in the choice of English words, he has usually fallen on Latin derivatives; on all which occasions his compositions read tolerably fair, and even his French *combativeness* may pass muster. Fortunately for him, though, his *philoprogenitiveness* is of *Greek root*, yet the word, united with IVE, is completely Latinized *(progenies)*. The term seems perfectly accordant with the force of the adjunct: this we shall explain, as it may assist our readers in their future progress.

By the etymology above admitted, and by the whole tenour of the work, it is evident that the doctrine consists in a power inherent in certain parts, which may impel the subject to perform certain acts. Yet the performance of these acts requires certain instruments. The hand is necessary for every kind of manipulation, but a good artist might, in a certain degree, direct another, and that other, by a facility of muscles, might perform the work better than his employer. Thus, in music, the ear is the instrument, but the faculty is that part of the mind by which certain sounds are found to harmonize best, or by which the truest melody is discovered. Hence it is found that, though the science of music cannot be comprehended by those who have never heard sounds, yet that the quickest ear is by no means the truest in musical sounds; that, though a man who has never seen, can never comprehend, the nature of colours, yet that the strongest sight will not always discriminate colours, nor feel that enjoyment from the mixture of them which others find. This is well illustrated in the formation of some animals.

"Many animals have the instruments to which certain faculties are attributed, but they do not produce the corresponding functions. Would it not be more natural to suppose that apes and monkies possess the building power on account of their hands, than to think that the beaver builds on account of its tail? Monkies have hands; they can put wood on a fire, but have they understanding enough to keep up the fire? According to this opinion, insects, craw-fish, lobsters, and especially cuttle-fish, ought to have exact ideas of extension, size, and mathematics, on account of their numerous and perfect organs of touch.

"The external instruments are often similar, and the functions performed by them quite different. There is a great variety of cobwebs, which different species of spiders make in order to catch flies. What diversity of structure in the nests of birds whose bills are similar! Animals of the same genus vary much in their food and their manner of living. The large titmouse builds its nest in hollow trees; the long-tailed titmouse in clefts of branches; the bearded titmouse among reeds; the titmouse of Poland suspends its delicate and curious nest on a slender branch; whilst the cuckoo, though it is endowed with a bill and feet fit to build, does not build at all. The hare and rabbit have similar feet, yet the hare lies in the midst of the fields, while the rabbit makes burrows."

The faculty, therefore, is that *vis* or force which makes a man feel a certain impression; and this impression will often exist without the power of completing the act. Thus, a person who has lost his sight, will call to his recollection an assemblage of colours which is most agreeable to him; and an animal not mutilated till after the organ of AMATIVENESS has been completely developed, will feel the stimulus of venery without the means of indulging it.

We have dwelt so long on this part of the subject, because to us it appears the most important difficulty attending former physical or metaphysical reasoning; and because, if the new doctrine is well founded, it solves this difficulty on principles perfectly consistent with sound physiology. We shall now offer our brief abstract.

In the introduction, we meet with many ingenious remarks on the study of psychology. The first part, on the Anatomy of the Nervous System, contains an epitome of the author's mode of dissecting the brain and nerves, not differing from what we have before noticed, but all directed to prove the great outlines of his theory; namely, the necessity of admitting as many organs as there are nervous masses; the proportion and relation between the grey and white substance in the nervous system; the different structure of the nervous parts of the abdomen and thorax; of the spine; of the five senses; of the cerebellum and the brain; the general principles of the commissures or unions of those nervous parts which are double. The communication, also, of the different nervous parts, is shown, especially of those which exert the greatest influence on each other. Hence, the conclusion is, that all the nervous parts are formed and perfected in the same manner, and not produced by elongation from the brain or spinal marrow. The physiology of the nervous system contains a very ingenious attempt to reduce subjects, usually considered metaphysical, to some order, and even to something like ratiocination.

nation. The author takes a view of the doctrine of innate notions, of education, of its effect, and of the passions. From various considerations, he shows that certain organs exist in different classes of animals, and in different animals of the same class; that some of these are larger and more perfect in some individuals than in others; that they are developed at different ages, and that their development may be facilitated, and their growth increased, by the manner in which they are brought into use, that is, by education and early habits.

In this part of the book, we have a very long, and to us it appears unnecessary, dissertation on fatalism and on materialism. Probably the meridian of Germany, particularly the catholical part, obliged the author to go so far out of his way as to quote scripture, and the fathers, in order to show that there is in some men a greater frowardness than in others; especially when he goes so far as to point out a conformation of the brain corresponding with these propensities. To us, nothing can be more philosophical, and, we will add, nothing more consistent with sound morality and true religion.

That there is a difference in the characters of children educated at the same school, and even under the same parents, can be no more questioned than that there is a difference in their features; nor can we dispute that there are such contrarieties in most characters, as oblige us to give a different description of each. If this variety depends on conformation, it will be said, where is the merit of a virtuous disposition, or how shall we condemn a vicious character? The answer is evident, that the virtuous character should be taught a due sense of gratitude to the Fountain of all Good for the happiness he enjoys; and the vicious commiserated, whilst he is warned against indulging passions which his own understanding will teach him must lead to misery and destruction. Thus the well-disposed may be taught modesty, and the wayward checked in their career. Should the propensities of the bad lead to acts destructive of the good order of society, and should they prove incorrigible, we have a written law to authorize us in *cutting them off from the congregation.* The mummery against penal laws is like most other parts of modern cant—nothing more than showing the abuse of necessary institutions. There are men to whom it is a charity to take away their lives, and who, till that service is performed, are the dread, and, by consequence, too often the hatred, of every lover of peace. The controversy concerning materialism is left just as it was and must ever be. The metaphysician may talk of what he cannot define,

 but but the true philosopher will confine himself to what he can demonstrate. That the soul is influenced by the body, cannot be questioned; and, unless we derive immateriality from revelation, we have no authority to use a term for what cannot be demonstrated. If we derive it from religion, it is no longer a subject of argument, but will be admitted by all who are satisfied with the evidence on which the truth of revelation rests. But surely these are not times to commit a Galileo to prison because he asserts, in contradiction to a language accommodated to our senses, that the world moves round the sun.

The succeeding chapter is devoted entirely to prove that all the moral sentiments and intellectual faculties depend, so far as the body is concerned, entirely on the brain, without any reference to the nerves proceeding from the spinal marrow, or to any parts of the abdominal or thoracic viscera. Many well-established facts and well-founded arguments are produced to show that lesions of the brain are constantly attended with alteration in the moral or intellectual character of the subject. Of those instances in which, by suppuration in man, and by mutilation in other animals, a considerable part of the brain is lost, it is remarked, that the senses have been destroyed corresponding with the parts destroyed or lost; but that, as none of the extremities of nerves were injured, organic actions would continue sufficient to preserve life for a certain time; and, if the parts were healed, life might be preserved with certain changes of character or privation of senses.

This chapter closes with some ingenious remarks on hydrocephalus, on ossifications of the brain, on the absolute and proportional size of the brain, on the facial angle, on the proportionate size of different parts of the brain, and of the mode of judging concerning such proportions. In most of these instances, Dr. Spurzheim shows the errors of former writers, and the insufficiency of the foundation on which their theories are built. The most important part of this chapter, in a practical point of view, is the section on hydrocephalus. In this the author asserts, that all the histories of chronic *hydrocephalus externus* are erroneous. In *hydrocephalus acutus*, the effusion may take place on every part where the inflammation exists; but that in the chronic it is always in the ventricles.

*The plurality of the cerebral organs* next engages our attention. It is first shown that the opinion of different parts of the brain destined to particular faculties, is by no means new; that philosophers and divines, even apostles, have shown the necessity of admitting either two souls or two distinct distinct properties in the human body; that some physiologists have even admitted the necessity of appropriating different portions of the brain to different faculties, as they saw different parts appropriated to different senses, though all acknowledged their incapacity to distinguish them. Several objections are then answered, some of which, we conceive, might have remain unnoticed; and a general conclusion is drawn, that it is necessary to make subdivisions of beings and functions, and that they have always been made, though the causes have not been discovered; that the proofs of every faculty, having its correspondent portion of the brain, is demonstrated by the various propensities of animals according with their cerebral formation; by the modification of the two sexes, corresponding with the difference of their sentiments and pursuits; and by the various effects of intense application, of dreaming, somnambulism, mental alienation; and, lastly, by the answers which the very objections to the theory have produced.

Having thus learned that the faculties are innate, that the manifestations of the mind depend on the brain, and of every particular faculty on some particular organ, we are next introduced to the author's method of *determining the functions of the cerebral parts,* or means of pointing out the organs of the manifestations of the mind. The first of these is *anatomy.* Here it is remarked, that nothing but actual experience can direct us; and that this is not different in the brain from the other parts of the body, as there is nothing in the sensible form of the stomach, kidnies, or liver, which would enable us to discover their properties. In the remarks on the comparative anatomy of the brain, the author makes free with his contemporaries, and most of all with Cuvier. This we are pleased to see, not only because that eminent physiologist is still living, but because his fame in comparative anatomy stands so high, that there cannot be a question of his readiness, as well as capacity, to answer, and thus that the truth must ultimately be established. A section follows on attempts which have been made to try the effects of dissecting out parts of the brain, which, according to this new system, are the organs of certain faculties. If we may judge by the passage, they have none of them confirmed the theory. This is accounted for by remarking, that the experiment, in order to be satisfactory, should not be confined to an horizontal section, but that the organ should be cut out perpendicularly to the bottom, by which process the nerves must be injured, and the animal killed. Even if the operation should be more successful, the injury done to the brain must interfere with the regular action of many many other parts of the brain, and, consequently, with the functions of many other organs. The following is given as the manner in which Drs. Gall and Spurzheim have proceeded in forming their conclusions.

"As the structure of any part does not indicate its function, and as the manifestations of the mind, nevertheless, depend on the organization, it must be examined on what other material conditions of any part its function depends. In every function, we may distinguish the energy or quantity, and the modification or quality. It is very difficult to examine the modifications; it is more easy to consider the different energy of the functions. It is then to be examined on what conditions the energy of the faculties depends. There is a general law, that the energy of the functions of any part depends on its size, and on its organic constitution; that is, on its extensity and intensity. It is also certain that, in order to judge the degree of activity of the faculties, it is necessary to consider, besides the extensity and intensity of the organ, the exercise of every faculty, and the mutual influence of the faculties upon each other. Now, among these conditions, the most easy to be observed is the size of the organs. Consequently, as the energy of the function depends on the size of the organs, and as the size of the organs is the most easily distinguished, it results that these means are the most proper for the discovery of the organs.

"There is, indeed, a general law throughout all nature, that the properties of bodies act with an energy proportionate to their size. A large loadstone attracts a greater mass of iron than a small loadstone. The fermentation of the same fluid is more energetic, if its quantity be more considerable. A great muscle of the same kind is stronger than a small one. If the nerves of the five external senses are larger on one side of the body, the functions are also stronger on the same side. Why should it not be the same in respect to the brain? Those, however, who object that we neglect the internal organization, are entirely wrong.

"In order to judge exactly of our inquiries, it must be considered that we do not endeavour to determine every degree of activity of any cerebral part, but only the nature of its functions, and to this end its size is sufficient. Gall, though he mentioned this difference in his lectures, was not careful enough to insist upon it. The internal constitution, though very important, is not easily distinguished. On account of its influence, however, we never compare the individuals of different kinds, not even the individuals of the same species, but, in order to conceive the first idea of any organ, we confine our observations to each individual in particular. I admit even the possibility that, in the same individual, the internal constitution of the different parts of the brain varies in the same way as the optic nerve may be more irritable than the auditory or olfactory nerve. It may, however, be observed, that a great difference in the size of the cerebral parts produces a difference in the manifestations of the mind. Indeed, the divers parts of the brain are differently developed: one is larger, another smaller; and, according to a general law, we are convinced that the functions of the parts, which are much developed, manifest themselves with more energy, while the small organs are less active. It results that, in persons endowed with partial genius, the organs are the most easily discovered. In fact, each organ has been discovered in persons who manifested one kind of function in the highest degree."

The author now feels it necessary to show how such properties of the brain are to be distinguished during life. This he explains by remarking, that the form of the skull is determined by its contents; which he proves by the marks from the convolution of the brain, by the loss of a part of the skull from the pressure of a fungus. This is, indeed, a question which will be readily conceded. But there is much greater difficulty in ascertaining the size of the brain, and still more in ascertaining the size of any particular organ. In answer to the latter, it is remarked, that most of the organs extend to, and consequently are manifested on, the surface, by the prominence each produces in the skull. It is, however, admitted, that many difficulties, some of which are insurmountable, attend this; and that, consequently, the science, in its present state, extends no farther than to designate such organs as are particularly prominent.

The seventh chapter contains the method pursued by these gentlemen of *pointing out the functions of the brain.* Here we are told not to expect any organ to point out those properties which are applicable to all the rest. Thus, we are not to expect an organ to demonstrate understanding, nor any of those common faculties connected with it, as perception, recollection, judgment, and imagination. These are called common faculties, being applicable to the others. For instance, in memory or recollection, it is a known fact that one person remembers names, another places, another events; in imagination or invention, that in one person it is confined to architecture, in another to poetry, in another to machinery, and in another to music. The same may be said of affections and passions used in a general sense. Hence it is observed, that the *general* configuration of the head is a very uncertain means of judging of the character; and that Gall himself, in the early part of his inquiries, fell into an error, which, for a long time, rendered him unable to satisfy himself, till he discovered, by slow degrees, the *separate* manifestation of some of the faculties.

The method pursued by these gentlemen was first to observe any peculiarity of form in an individual who was celebrated for any particular quality or faculty. If similar prominences minences appeared in several whose faculties are similar, this led to a suggestion that an organ was discovered. The inferior animals were carefully examined, and whatever instinctive or innate properties they possess, induced a comparison of the formation of certain parts of the brain with men who were eminent for similar endowments. Even the signs made during certain mental sensations were attended to, an advantage more easily acquired on the continent, where every conversation is so much mixed with pantomime. The skulls also of different nations are said to have assisted the inquiry. The Chinese exhibit most strongly the faculty of colours, and the Kalmucs that of coveting, which leads to stealing.

After these remarks, and introductory to the manifestation of particular faculties, we have a chapter *on the particular organs.* In this it is remarked, that there are certain properties common to all the senses; that the organs are double. Here we are told that the habit of using the right side more than the left is original, and not acquired; that the right eye, hand, and leg, are the largest in most people, and also the right breast, and the right hemisphere of the brain. Without disputing the general result of these observations, we cannot help observing, that in most females the left breast is largest, and the left cheek the fullest. This must have come within the observation of every experienced surgeon who has been often consulted for complaints in those parts, and is probably imputable to the habit with most people of sleeping on their right side, the frequent pressure on which may induce absorption of the contents of the cellular substance. Probably Dr. Spurzheim may have drawn his conclusions from the state of childhood, before such an effect can be produced.

Though these organs are double, it is remarked, their consciousness, or the effect of the impressions produced on them, is single. The various modes by which philosophers have endeavoured to account for this, are detailed; and the author, after objecting to each, concludes with remarking, that we are still unacquainted with the true cause. Every sense, it is next remarked, has its own nature, or rather its peculiar faculty;—every sense may be exercised, and is, for the most part, strengthened by exercise. Many other observations follow on the external senses, all tending to show that they are each regulated by nerves, which can receive no other impressions than such as relate to their own peculiar faculties; and that they have parts of the brain to which they convey their particular impression. This, with some other other observations, introduces the description of the manifestations of the various organs, as marked on the diagram.

The first of these is the organ of *amativeness*, or love for the other sex (see I.) This organ is discovered in the space between the mastoid process behind the ear, and the protuberance of the occipital spine about the middle of the neck. By its situation, it increases considerably the volume of the neck, which is always, if we may believe our author, particularly tumid at certain periods. To this peculiarity he imputes the greater thickness of neck in entire animals above those who are castrated; and several passages are cited from the ancients, as well poets as philosophers, to show the sympathy of the neck with a strong propensity for the sex.

The organ of *philoprogenitiveness* (II.) we shall transcribe, to show the manner in which some of these discoveries have been made, and the foundation on which they rest.

II. *The order of philoprogenitiveness, or love of offspring.*

"I shall endeavour to prove by reasoning, according to the method mentioned above, that it is necessary to admit a particular organ of philoprogenitiveness: I shall afterwards state the circumstances which led to its discovery. In some kinds of animals, neither male nor female take care of their progeny; the eggs are resigned to chance, and to some external influence: this is the case with insects, reptiles, and fishes. Even among birds, the cuckoo gives a striking example of it. This bird has a great propensity to physical love, but it neither builds a nest, nor hatches its eggs: it deposits its eggs in the nests of other little birds which live on insects, placing only one egg in any individual nest; and the other birds hatch and nourish the young cuckoo with particular attachment. In some other kinds of animals, the females alone take care of their progeny. Bulls, stallions, dogs, cocks, &c. are indifferent about their young; while the cow, mare, bitch, hen, &c. are extremely attached to them. In other animals, again, the males and females form an attachment for life, and both sexes take care of their progeny; this instinct, however, is more energetic in the females. The fox, which so much resembles the dog, differs from him in this respect. The fox is attached to its female for life, and partakes of the same cares with her; and if the female be killed, he seeks for food for his young ones. Philoprogenitiveness, however, is stronger in the female than in the male; for, if they be pursued, the male leaves the young ones sooner than the female. Many birds are paired, and both males and females take care of the young. These differences are constant: does not each of them, even on the slightest consideration, seem to require peculiar organization?

"In the human race, this propensity is, in general, stronger in women than in men. This difference is not only perceptible between fathers and mothers, but also between the sexes in general.

A male servant seldom takes care of children as well as a woman. Besides, this difference is not only sensible in grown-up people, but even in children. Present to children playthings indiscriminately, boys will choose horses, whips, drums, &c.; girls, on the contrary, will prefer dolls, cradles, ribbons, &c.

"In every species of animals which take care of their progeny, there are some females which do not feel this propensity, while certain males of these kinds excel in this inclination. Even certain women consider children as a heavy burden, others as their greatest treasure and happiness. This is not only the case among wretched persons, but indiscriminately among rich and poor, among persons of good and bad breeding. In general, all the proofs which have been adduced of the plurality of the organs, may be applied to the organ of philoprogenitiveness in particular. Therefore its existence is necessary.

"Dr. Gall observed a distinct protuberance on the posterior part of the skulls of women (Pl. VII. fig. 1. II.*); and in comparing the skulls of his collection, he found a similar elevation on the skulls of children and on those of monkies. Consequently, it was necessary to point out a faculty common to all of them. During five years he was occasionally occupied with this consideration. He thought, for some time, that it might indicate the greater irritability of women and children; but this supposition he did not long entertain, because irritability is a common quality of every organ. He was in the habit of suggesting his difficulty, relative to this protuberance, to his auditors; and a clergyman, who attended him, observed, that monkies have much attachment to their progeny. Gall examined this idea. In fine, he found that this protuberance, which is situated immediately above that of physical love or amativeness, and corresponds with the general protuberance of the occiput, is the organ of philoprogenitiveness. The development of this cerebral part always coincides with the energy of this propensity. Species, sexes, individuals, which are endowed with a great deal of maternal love, have this organ much developed. In women and females, this organ is, in general, larger. Gall possesses the skull of a woman who, being sick, had the confirmed notion of being pregnant with five children. The corresponding organ in her is extremely developed. There are nations which excel in this propensity, and the development of the respective organ is proportionate. Negroes manifest this propensity in an eminent degree, and this organ is much developed in them. These are facts which every one may verify.

"It is objected that love of children is the result of moral sentiments, of self-love, or of the desire of suckling, and not at all of a

* The last number in each reference to the plates corresponds with the figure on the diagram. The recollection of this will be found very useful in perusing the work, because there are several inaccuracies in the references to the plates, which may, for the most part, be corrected by always keeping these numbers on the diagram in view.

particular particular propensity. These causes, so commonly admitted, cannot produce love of offspring; for in many animals which love their progeny, these causes do not exist. No animal, below man, has any idea of duty or religious sentiment: birds do not give suck, yet they love their young. This is also the case with men and males in general. Many mothers do not give suck, and yet they love their children much. Moreover, in mothers there is no proportion between moral or religious sentiments, and philoprogenitiveness. Consequently we must admit a particular organ for this propensity.

"I have already mentioned that in mankind, and in those species of animals where females take care of the progeny, certain individuals are quite indifferent to their progeny.—(Pl. VII. fig. 2, II.) In mankind this phenomenon must be considered as an indirect cause conducive to infanticide. We have examined the shape of the head in twenty-nine women who were infanticides. Twenty-five of them had the organ of philoprogenitiveness very small. The want of this organ does not excite a mother to destroy her child, but a mother destitute of this propensity is less able to resist those external circumstances which provoke her to commit this crime. Such a mother will not resist as much as she would have done if her mind had been influenced by the powerful energy of philoprogenitiveness.

"The aim of this faculty is obvious—care of the progeny; its activity may be too energetic, and do harm to children in spoiling them; and if it be very small, there must follow indifference about progeny. The protuberance which indicates the development of this organ, placed in the posterior lobes of the brain, is commonly single, although the organ is double, that is, one on each side. It is single when the posterior lobes of the brain are very near to each other, and double when the posterior lobes are somewhat separate. This difference of form is common to all organs situated in the mesial plane of the head.

"By this and the preceding organ, it is very easy to distinguish the skulls of males and females of the same kind, and, consequently, those of men and women. It is peculiarly worthy of notice, that, throughout all animals, a striking similarity is preserved in the skulls of both sexes. The skulls of men and males are generally shorter and larger, those of women and females longer and narrower."

We shall next offer an extract from the account of "*the organ of the propensity to build, or constructiveness.*"

"Gall observed that those who had a particular disposition to mechanical arts, presented a face of somewhat parallel form, that is, a face as large at the temples as at the cheeks; consequently, that a greater disposition to mechanical arts is indicated by the development of the brain at the temples.—(Pl. XIII. fig. 2. VII.) He found this sign in great mechanicians, architects, sculptors, and designers. The skulls of animals which build, and those of others which do not build, present a remarkable difference at the place

 where where this organ is situated; for instance, the skulls of rabbits and of hares. It is known that rabbits build burrows, while hares, which in general resemble rabbits, lie in the field. In the beaver, marmot, field-mouse, &c. this organ is distinctly expressed.

"A certain skull is preserved at Rome, which is said to be the skull of Raphael. There exists some doubt of its reality. Professor Schell, of Copenhagen, brought a cast of it in plaster to Gall, and asked him his opinion relative to this skull. Gall answered that three organs were very considerable; that the organ of mechanical arts was more developed than he had ever seen it before; and that the organ of imitation, and that of physical love, were very large. Gall possesses the skull of a milliner of Vienna, who had a good taste, and understood perfectly to change the forms of her merchandises. In this skull, the organ in question is prominent.

"Adversaries of our doctrine may ridicule a comparison between Raphael, a milliner, and a field-mouse. They may laugh at a doctrine which, as they conclude, attributes to a similar organ the sublime conceptions of Raphael, the pretty productions of a milliner, and the inartificial habitation of a field-mouse. But does not the sloth creep by means of organs similar to those by means of which the horse can gallop, or the roe run? Does not the ass cry by the organs by means of which a Catalani sings? It is, indeed, true, that this faculty alone does not produce the sublime conceptions of Raphael, but it was essential to the execution of their objects."

The account of the succeeding organ *(covetiveness)* is extremely interesting, from the very curious anecdotes with which it abounds concerning "persons who have a particular propensity to steal." Though none of our readers, we conceive, can be entirely unacquainted with such unfortunate characters, even in that class of society which renders the practice as unnecessary as it is disgraceful and dangerous, yet we shall copy a few sentences to show that one unhappy accomplishment is not peculiar to our fair countrywomen.

"Certain persons have a particular propensity to steal or rob. It is known that Victor Amadeus I. King of Sardinia, took every where objects of little importance. Saurin, Pastor at Geneva, though acquainted with the best principles of reason and religion, was overcome continually by the propensity to steal. Another individual, of good breeding, was given up to this inclination from his infancy: he betook himself to the military service, in hopes of being restrained by the severity of its discipline; but, as he continued to steal, he was in danger of being hanged. Struggling against this propensity, he studied theology, and became a Capucin: his propensity followed him into the convent, but he took only little things, as candlesticks, snuffers, scissars, drinking-cups, and glasses. He did not, however, conceal the stolen objects, but acknowledged that he had taken them home that the proprietors might have the trouble of carrying them to their houses again. A

person person employed by the government of Austria, and established at Presbourg, had filled two chambers with stolen furniture, but he never dared to make use of it. The wife of Gaubius, the famous physician at Leyden, had, in so high a degree, the propensity to steal, that, when making purchases at shops, she always endeavoured to take something away. Her husband ordered a servant to follow her, and to prevent or to compensate for her theft. The Countess M***, at Wesel, and J***, at Frankfort, manifested the same propensity. Madame de N*** had been educated with great care; her understanding and talents assured to her a distinguished place in society; but neither her breeding nor education secured her against the powerful propensity to steal."

These anecdotes are continued for several pages, and describe men of all ranks and professions who were incapable of resisting the propensity.

We have transcribed the following, to show the manner and degree in which one faculty may be modified by, or is absolutely inconsistent with, certain others.

XIII. *Organ of benevolence in man, or of meekness in animals.*

"For a long time Gall did not think of placing goodness of heart in the brain. A family at Vienna often praised the goodness of one of their servants. This family several times told Gall, that he ought to mould this servant in plaster. At last he actually did so, and observed a considerable protuberance on the superior middle part of his forehead. This organ was afterwards confirmed by numerous observations; for it is very easy to examine and verify this organ in children and in adult persons.—(Pl. VII. fig. 1; Pl. XI. fig. 1. (XIII.)) This organ may also be proved by reference to animals, either in comparing different species, or different individuals of the same species. Several kinds of animals are naturally meek, as the roe, goat, sheep, while others are wild, savage, and mischievous. Some dogs, horses, cows, &c. are meek and familiar, while other individuals of the same kind bite, kick, &c. The mild and good-natured animals have the place of their forehead corresponding to the organ of goodness in man, elevated and prominent (Pl. X. fig. 1 and 3, XIII.), while the ill-natured present a hollow at this place (Pl. X. fig. 2 and 4, XIII.).

"It is sometimes maintained that goodness is the result of the want of courage. But it is in my opinion a law, that the want of any faculty cannot produce any positive sentiment. On the other hand, many persons are very quarrelsome, and at the same time very good-hearted. In the same way, active cruelty cannot be the result of the want of goodness; for cruelty is a positive sentiment. It is true, goodness or compassion cannot exist in cruel beings which are fond of tormenting others, but cruelty belongs to the organ of the propensity to destroy, without being restrained by any other sentiment.

"This faculty, although it exists in animals, is greatly magnified and ennobled in mankind. In the greatest number of animals it is is restrained to a passive goodness, but in man its sphere of activity is very considerable. It produces in man goodness of heart, kindness, peacefulness, mildness, benignity, benevolence, complaisance, clemency, mercifulness, compassion, humanity, hospitality, liberality, equity, cordiality, urbanity, in one word, *Christian charity.*"

We shall pass over the enumeration of any other manifestations. Those we have given are sufficient, as specimens; and such of our readers as feel a greater interest in the subject, will peruse the original. At the close of each chapter a sort of recapitulation is given. The following apologetic sentence is all we shall offer for the present.

"A double objection against this kind of considerations is made. Some adversaries object that there are too many organs; others say, that there are not enough. Those who find the organs too multiplied, must know that every organ is admitted by the same proofs, namely, by those which establish the plurality of the organs, and that it is verified by experience. The independence of one organ is neither more nor less certain than that of any other organ; and, if any proofs be admitted in respect to one organ, they must be agreed to in respect to all other organs. Those who think that we do not admit organs enough, must consider that every faculty may be applied to an infinite number of things; for instance, seeing is always seeing, but what an infinite number of objects may be seen! Hearing is always hearing, and so on as to every external sense. It is the same with the internal faculties. Constructing is always constructing, but what an infinite number of objects may be constructed, &c. Moreover, it is to be observed that a great number of actions (not a great number of faculties) result from the combination of different faculties; and therefore it is not surprising to observe so many effects produced by so small a number of them."

"The different modes of action of the special faculties of the mind" are next considered. In this chapter the author shews, that many faculties are considered as different, which are only different degrees of the same faculty; that others are modified by impressions derived from other faculties. Hence the vast variety in the human character, and the apparent contradiction in many; and hence it is that we rarely see the most depraved entirely destitute of some good qualities, nor the best without their failings. This leads to a consideration of "the mutual influence of the faculties of the mind as they concern the morality of our actions." Here the author distinguishes between those faculties which are active, and those which are auxiliary. Of the former are hunger, love, propensity to fight, build, or accumulate. These should be directed by the faculties more connected with mind; and this law of subordination of the faculties, leads immediately to the consideration of moral good and evil.

evil. That physical and moral evil really exists, one should have thought it unnecessary to prove. That they are both consistent with the free agency of man, we know not how to deny; but there will be always a difficulty in explaining the causes of evil, and we do not perceive that Dr. Spurzheim has done any thing towards elucidating so dark a subject. It is enough to say, that he has not involved it in greater obscurity, or, in other words, that, as no one ever doubted a propensity in most of us to indulge sensuality to excess, so no one can question, without ever referring to the new doctrine, that these faculties or propensities are in some particularly powerful, and that too often they are not controlled by the auxiliary or directing faculties.

The remaining chapters are—on *the modifications of the manifestations of the mind.* Under this head are considered association of ideas and mnemonics.—The *mutual influence of the faculties as the cause of different characters and talents.*—The *difficulty of judging the actions of other persons.* Here the invention of arts and sciences is considered, and also the necessity of some indulgence to the failings of others. The concluding paragraph of this division is so well disposed, that, though it contains nothing very new, we shall make no apology for transcribing it.

"From the modifications of our faculties results still a very important practical rule—indulgence. It is impossible that other persons should feel and think in every point in the same manner as we do. In the same way, as it is generally admitted, that the functions of the five external senses cannot be altogether the same, and without any modification—as it is proverbially said, *De gustibus non est disputandum*, the internal faculties are also modified, and no one has a right to desire other persons to feel and think in the same manner as he does. A certain indulgence is necessary in society. I do not maintain that every manner of feeling and thinking, and every action, can be tolerated. There is a common touchstone for every individual. The feelings, thoughts, and actions, must be conformable to the absolute conscience of man; all other modifications ought to be tolerated. This principle may be applied to both sexes, to all conditions and ages; and no friendship can be permanent without indulgence as to many modifications in the manner of feeling and thinking. It is the same in religious and other opinions. St. Paul said to the Romans, 'One believeth that he may eat all things; another, who is weak, eateth herbs: let not him that eateth despise him that eateth not, and let not him that eateth not judge him that eateth. One man esteemeth one day above another, another esteemeth every day alike. Let every man be fully persuaded in his own mind. We then that are strong, ought to bear the infirmities of the weak, and not to please ourselves. The kingdom of God is not meat and drink, but righteousness and peace.'"

A chapter

A chapter on *sympathy and antipathy* might, we conceive, have been omitted. What is admissible in it is not new. That on *pathognomy* contains many new and judicious rules for judging of the characters of others. The physiognomy of Lavater is shown to be unsatisfactory, inasmuch as he measures only the whole head, thus overlooking the individual faculties, which are considered by the author as producing all the variety of character. For the *general* principles of pathognomy, the author insists principally on the corresponding actions of the whole body, or of all the parts, particularly the features. This is followed by some well-digested remarks on the impression of pain or pleasure produced by the presentation of various objects, and on the compound effect, according as two or more different faculties may be affected at the same time.

The remarks *on man as an object of education, and correction or reform*, are such as might be expected from the whole of the preceding work. The author regrets, with much truth, that we know so little of man; but urges that he should be studied most of all for the above purposes. In education, the prevailing faculties should be well ascertained, and a direction given so as to improve the best by continual exercise, and repress the bad, either by entire disuse, or by constantly restraining its action whenever it is excited. The same should be the aim and constant employment of the legislature.

The last chapter is by far the most important of all, and we scruple not to say, that it may prove a sufficient apology for the tediousness attending the tautology of some others. It is *on the diseased state of the brain, and on the derangements of the manifestations of the mind.* This, of course, leads to the consideration of lunacy, particularly the diagnosis of the immediate or proximate cause, as far as experience in morbid dissections, compared with the symptoms during life, may direct us. It would ill become us to give an opinion on this subject. We pretend not to have sufficiently improved the opportunities of examining the brain of these subjects ourselves, and unfortunately there is too great a deficiency of records to direct us. Why have we no information from men who profess to devote their lives to such cases, and who have ample opportunities of examining the brain, from the facility with which such dissections are concealed, where it is necessary or desireable? If it should be answered, that hitherto dissection has taught us little, let us at least know that little, and let us be informed of the number of cases in which nothing has been discovered. From this time, we trust, the improved mode of dissecting the brain, brain, which seems generally approved, will be adopted, and that facts will be recorded.

Such is our epitome of this truly novel production. If we have said but little of its merits, it is not from any wish to undervalue it, but because we conceive ourselves hardly capable of forming a true estimate of it. That it contains much valuable matter is certain; and if in other parts it is less so, and even in some instances erroneous, we shall still for ever respect the industry of men who have discovered a new science, and taken pains to make it generally known.

*Explanation of the Diagrams on the Plate referring to the various Organs.*

"I. Organ of amativeness *(physical love)*.

II. ——— philoprogenitiveness *(love of offspring)*.

III. ——— inhabitiveness.

IV. ——— adhesiveness.

V. ——— combativeness.

VI. ——— destructiveness.

VII. ——— constructiveness.

VIII. ——— covetiveness.

IX. ——— secretiveness.

X. ——— self-love.

XI. ——— approbation.

XII. ——— cautiousness.

XIII. ——— benevolence.

XIV. ——— veneration.

XV. ——— hope.

XVI. ——— ideality.

XVII. ——— conscientiousness.

XVIII. ——— firmness or determinateness.

XIX. ——— individuality.

XX. ——— form.

XXI. ——— size.

XXII. ——— weight.

XXIII. ——— colour.

XXIV. ——— space.

XXV. ——— *order?*

XXVI. ——— *time?*

XXVII. ——— number.

XXVIII. ——— tune.

XXIX. ——— language.

XXX. ——— comparison.

XXXI. ——— causality.

XXXII. ——— wit.

XXXIII. ——— imitation."

### *Medico-Chirurgical Transactions, Volume the Fifth.*

(Concluded from p. 420.)

*On the Treatment of Erysipelas by Incision;* by A. C. HUTCHINSON, M.D. Surgeon to the Royal Hospital, at Deal.

No one at all conversant with the diseases of seamen can be ignorant of the great ravages attending erysipelas, especially in the lower extremities. Gangrene and deep-seated suppuration, with all its consequences, frequently render incisions absolutely necessary in an advanced stage of the disease, in order to dislodge matter which is insinuating itself into every part of the cellular membrane. Mr. Hutchinson found great advantages from early incisions, in addition to the general treatment, which was of course governed by circumstances. The paper is well written, and the success of the author's plan entitles it to general consideration, particularly in that line of practice in which these cases are most common.

*Case of Obstructed Aorta;* by ROBERT GRAHAM, M.D. one of the Physicians to the Royal Infirmary at Glasgow.

This is a most interesting and important paper. A boy, after the common symptoms of pneumonia, expectorated matter, and had subsequently considerable palpitations, but without any purple appearance on the skin. By proper remedies, all the symptoms subsided, and the patient left the hospital. In about six weeks, he returned with palpitation, dyspnoea, and a throbbing of the carotid and subclavian arteries. These symptoms increased for about three months, when the patient expired. The pulse, during his last residence in the hospital, fluctuated from 90 to 116, but was always regular, with different degrees of firmness and strength. On examining the body, the principal thing deserving notice was an unusual expansion of the aorta near its origin, so as to form a kind of pouch, but, after giving off its branches to the head and superior extremities, its diameter was preternaturally contracted.

"It was continued of this diminished size, till after its union with the *canalis arteriosus*, where it was completely impervious. The coats were not thickened, or in any way diseased, except that, about half an inch below the stricture, there was a smooth elevation on the inner surface, less raised, but having nearly the diameter of a split pea; otherwise the appearance was exactly such as if a ligature had been tied tightly round the artery. The obstruction was about a line in breadth. The artery then received three trunks about the size of crow quills, and near them three smaller ones, afterwards resuming its natural size along the *vertebræ*. These three three trunks are evidently the uppermost of the inferior intercostals. Their coats were remarkably thin, like those of veins. A probe passed from the pulmonary artery along the *canalis arteriosus*, to the obstructed portion of the aorta, but from its thickened appearance it did not seem probable much communication by means of it could have been allowed, and the florid countenance of the boy during life establishes the same conclusion. There having been no suspicion of this singular deviation from the natural structure, till after the contents of the thorax were removed from the body, it was impossible to trace with the accuracy that could be wished, the anastomosing branches by which the circulation had been carried on in the inferior parts of the body; but I think enough has been observed to lead us very near the truth. The *arteria innominata*, the left subclavian, the superior intercostals, and the mammary arteries, were much enlarged. The epigastric was reported of its natural size. These facts, and the aorta acquiring at least very nearly its natural size immediately below the stricture, shew that the blood did not pass to the inferior extremities, in any material quantity, as might perhaps have been expected, by the inosculations of the mammary and epigastric arteries, but chiefly by the communications of the superior intercostals and the mammary arteries, with the three large branches entering the aorta below the stricture: also from the mammaries and thoracics through others of the intercostal and diaphragmatic arteries."

Some judicious remarks follow. The writer conceives, with much propriety, that the preternatural appearance in the aorta was not original, but the effect of disease; that the enlargement and consequent stricture had been gradual; and the violence done to so important a blood vessel, shews how considerable the resources of nature are to protract life under circumstances the most unfavourable even to the circulation itself.

*On the Diuretic Properties of the Pyrola Umbellata;* by W. Somerville, M.D. Deputy Inspector of Military Hospitals, &c.

It is too common an opinion, that the *Materia Medica* is much too considerable already. This is a dangerous prejudice, when we recollect the vast variety in the human constitution, and the difficulty of suiting remedies to each. This is exemplified in no class of remedies so remarkably as in diuretics. Hitherto, we fear, the valuable addition described by Dr. Somerville has not been naturalized in England; the extract, however, has proved equally efficacious.

*Case where a Seton was introduced between the Fractured Extremities of a Femur, which had not united in the usual Manner; with some Observations on the Methods which*

*have been employed to produce re-union in Fractured Bones.* By JAMES WARDROP, Esq. F.R.S. Ed.

This is a judicious improvement in the mode of treating broken limbs, which, from various causes, instead of uniting by callus, have only smooth surfaces by the absorption of the rough edges at the fracture. The cases are well described, but not easily explained without the accompanying plate.

*Further Observations on the Diseases which affect the Synovial Membranes of Joints;* by B. C. BRODIE, Esq. F.R.S. &c.

This is an ingenious dissertation, but, in our opinion, is calculated for a society, where opinions are discussed. Had the cases been minutely recorded, with no other remarks than such as naturally arose from each, and such as would lead the reader to the practical inferences, we should have thought the whole better suited to the plan of the society, and the class of readers for whom the Transactions are intended.

*Case of Aneurism of the Glutæal Artery cured by tying the internal Iliac;* by W. STEVENS, Esq.

We cannot make the same objection to this as to the last article, nor can it be necessary to detail the case in order to shew the merits of the author. If this is not the first time so bold an operation has been attempted, it is, at least, the first time it has succeeded. The following remarks, however, are selected to show how much, and with what advantage, the operation for aneurism may be simplified.

"In performing this operation, I used only one ligature, because I believe that one ligature is perfectly sufficient for any artery. When we expose the iliac or any other great vessel, it is necessary to separate the artery from its surrounding connexions, only for a small space: a space no larger than just sufficient to allow the insinuation of the fore-finger, behind it. With the point of this finger we raise the vessel, and examine it: with a small blunt or aneurism needle, pass a ligature behind the artery, draw it to the upper part of the vessel, where it is surrounded by its cellular substance, nourished by its vasa vasorum; and tie it there. When we tighten the ligature, the opposite surfaces of the internal coat are brought into immediate contact; the ligature wounds the internal coat and thus excites inflammation. When the internal surfaces are inflamed, and in contact, they adhere to each other, and then only is the patient out of danger.

"It often happens in aneurism, that the artery, even at a distance from the tumour, is so completely ossified, that it will not inflame, and consequently not adhere; the ligature soon comes away, and is followed by a secondary hemorrhage.

"Though

"Though we have a ligature beneath this part, it certainly can do no good; it cannot prevent the hemorrhage, as the bleeding is nearer to the heart.

"The vile practice of insulating a large portion of an artery, and then only half tying it; the use of the four ligatures, and other unhappy contrivances, have been very justly and very happily criticised by Mr. John Bell;—but even Mr. J. Bell uses one ligature too many: he, like Mr. Abernethy, and many surgeons of the present day, ties the artery with two ligatures; and cuts it across betwixt them.

"By placing the artery in the same situation, that it is in on the face of a stump, it was expected that secondary hemorrhage would occur as seldom after the operation for aneurism, as it does after amputation; the circumstances, however, are very different: in cases which require amputation, though every other part of the limb may be diseased, the arteries are generally healthy; and when properly tied they seldom bleed. In aneurism, though every other part of the system is healthy, the arteries are generally diseased. It is this diseased state, this premature old age of the arterial system, which is the cause of aneurism, and of the hemorrhage which is so frequent and so troublesome after the operation.

"If hemorrhage, after the operation for aneurism, is produced, not by the position of the artery, but by the diseased state of the arterial system, the double ligature can do no good. If an artery is diseased, the lower ligature cannot make it healthy: though it has retracted, it will not adhere; though we have two ligatures, they cannot prevent the secondary hemorrhage.

"The surgeon who uses the two ligatures gives himself unnecessary trouble, his patient unnecessary pain, and leaves three extraneous bodies in the wound, while one only is necessary; the lower ligature insulates a portion of the artery; this insulated part remains in the wound as a foreign substance, so does the lower ligature; in truth, this lower ligature can do no good by remaining in the wound; irritating and keeping it open, it does much mischief.

"Even in aneurism, the artery where we operate is sometimes healthy; when such an artery is properly tied, we have nothing to fear from secondary hemorrhage; its cavity is soon obliterated by adhesion; and the natural position of the vessel is perhaps more favourable for this process, than when the artery is retracted.*

"This was the only case of aneurism that I either saw or heard of during a residence of nearly four years in the West Indies. In

* "I express my disapprobation of the double ligature, because I believe it to be a bad practice, a practice that is radically wrong, and has nothing to recommend it but the respectable names of Mr. John Bell, Mr. Abernethy, Mr. A. Cooper, &c. No one can value these gentlemen more highly than I do; every surgeon must feel indebted to them for the good they have done to the profession. As an individual, I feel grateful to these gentlemen for much personal kindness."

St. Croix, St. Vincent's, St. Kitt's, and some other islands, so seldom is it met with, that I know practitioners, who, during thirty years' extensive practice, have never seen a case of aneurism, stone, or any other disease produced by the deposition of calcareous matter."

Some questions follow concerning the cause of aneurism. As they throw no light on the subject, we shall not transcribe them, but leave the author in full possession of all the honour he may justly claim, for the boldness, success, and originality of his operation.

---

*Practical Observations on Necrosis of the Tibia; illustrated with Cases, and a Copper-Plate. To which is added, a Defence of a Tract, entitled, "Description of an Affection of the Tibia induced by Fever," &c.* By THOMAS WHATELY, Member of the Royal College of Surgeons in London. 8vo. pp. 130. Callow, 1815.

THE author, some years ago, published an account of an affection of the Tibia after fever, which had escaped the observation of the profession as distinguished from necrosis. The characteristic excellence of Mr. Whately's writings, is a sound exposition of plain practical facts; and his former pamphlet having led his mind towards the subject of Necrosis, he has, in the present little work, given a good history of the disease and its treatment after his own manner. Some remarks also, which his former publication drew from the reviewers, have contributed to keep the subject on the mind of the author, and have been, perhaps, remotely the cause of the present book; if so, neither he nor the public will regret them. The former part of the work consists of a history of the approach and early symptoms of Necrosis, which are detailed in the following passage.

"In those cases of Necrosis on the Tibia, which have fallen under my own observation, pain has suddenly seized the bone, without any previous indisposition whatever; (a slight injury to the part has, however, in several instances been sustained.) The pain was soon followed by an increase of heat, and swelling of the limb, attended with violent inflammation. All these symptoms have rapidly increased, and to a very alarming degree, almost always confining the patient to bed, and terminating at length, in about a fortnight, or three weeks, in a large deep-seated abscess in the course of the Tibia; which has either burst, or been discharged of its contents by a lancet. This has usually been followed by four or five others, or more, at different periods afterwards; all of them for the most part situated in the course of the Tibia, or connected by sinuses with it.

"An attack of Necrosis on the Tibia is therefore an attack of inflammation, followed by suppuration, and producing certain effects effects upon the bone. It will be of use to explain the action of this inflammation, and its consequences, as I do not think these points are quite so well understood as they ought to be. By an attention to the history of cases of Necrosis Tibiæ faithfully related, it would appear, that the inflammation first attacks the substance of the bone; and as in that of the soft parts, where we can more accurately trace its progress, is unquestionably more or less violent, or to a greater or less extent, in different cases; that is to say, the whole, or a part only of the Tibia, may be affected by it; thereby producing considerable variation in its consequences. It appears, however, that in the greater number of cases, the disease extends over the whole of the Tibia. It may seem strange to some, that a bone so hard and solid, which appears to have so few red blood vessels in its composition in a natural state, should be the subject of high inflammation. The fact however is certain; and it will be clearly shewn, that the whole of this bone is frequently destroyed by its action, and removed from its place in the system in a very short time."

The treatment of the author is chiefly peculiar in this, that he recommends the early extraction of the dead portion of bone, while the new osseous matter is so soft as to allow of this operation being performed without great difficulty.

"As soon as he has recovered sufficient strength, *(from the preceding inflammation,)* the pieces of bone should be extracted by the forceps; enlarging the openings where it may be necessary by sponge tent; after which, a complete cure will ensue. But, if any of the latter effects take place, the utmost attention should be paid, and, as early as possible, to the removal of the dead bones. This is, however, a nice, and often a difficult task, and will, in many instances, require skill and manual dexterity, as well as length of time to accomplish. As soon as any portion of the bone, greater or less, is found to be completely separated, which in most cases is known by its becoming moveable, it should be extracted, if the patient's strength will admit of it, and it can be done with safety. There are two principal advantages attending this mode of procedure, viz. the new or adventitious bone will be softer, and therefore more easily acted upon by any instrument that may be necessary, should it occasion any impediment to the removal of a sequestra; and, at this period of the complaint, it will embrace or hem in the sequestra in a less complete and firm manner. The irritation occasioned to the constitution as well as to the part, by the presence of a foreign body, will likewise be the more speedily removed, and the recovery of course much expedited. When the whole cylinder of the dead Tibia becomes surrounded by the new bone, forming a complete sequestra, and no portion of it is protruded through a vacancy in the new cylinder, more skill and address will be required to remove it, by an operation, than in any of the other varieties of this disease."

The latter part of the book contains a good parallel of the [illegible] two two complaints which have been the subjects of the author's consideration, and the material circumstances, history, and treatment, in which they differ from each other. In Necrosis the author thinks that we expect, or have trusted, too much to nature for its cure,—"Too much is in general left to nature;" and, although we should be apt to combat this tenet as one of general application, we believe the interference and decisive operation of art is necessary to remove the morbid exfoliation. The mode of doing this in Necrosis is by the application of mechanical force, according to the nature of the case, (for no two cases are alike;) and, in the affection of bone after fever, Mr. W. advises the use of caustic. The time which is required for the separation of bone destroyed by kali purum, is about three weeks or a month, and the small piece of dead and detached bone will be seen under the sloughed portion. We have had an opportunity of witnessing the correctness of these remarks of the author, as well on this as on other parts of his subject; and we take our leave of this useful essay, which further contains many cases and drawings in illustration of the doctrine which he inculcates, by the following quotation of one of the former.

"Case II.—Elizabeth King, No. 31, Red Cross-street, Union-street, Borough, aged thirteen years and a half, having had no previous fever, or indisposition of any kind, was seized in August, 1807, with violent pain in the right leg, extending in the course of the Tibia, from the knee to the foot. It swelled and inflamed the same day; she passed a very bad night; and the following day all these symptoms were much more violent. In about ten days, suppuration took place; the abscess, situated about the middle of the Tibia, was immediately opened, and about a quarter of a pint of matter discharged. Even after this, the pain, inflammation, and swelling, continued very violent, and a large quantity of matter was daily discharged. She continued nearly in this state about a month longer, when the violence of these symptoms in some degree abated. During all this time she was confined entirely to her bed, and had very little remission from pain night or day. Soon after this period, another abscess formed in the course of the Tibia, at some distance from the former, which burst, and discharged a large quantity of matter. She was confined to her bed for three months longer, during which time three more abscesses formed along the Tibia; two of which were opened, and the other burst. These abscesses were situated in different parts of the leg, from a little below the knee nearly to the inner ancle. All the violent symptoms now gradually abated, and in a short time the common integument, covering the Tibia at its upper part, ulcerated away; by which a considerable portion of that bone became exposed. About two months afterwards, being being about six from the attack, the girl was in a very deplorable situation; being extremely weak and emaciated, unable to put her foot to the ground, and walking with great difficulty with the assistance of crutches. Almost the entire cylinder of the Tibia, to the extent of four or five inches from the knee, became elevated from its natural situation; apparently by the pressure of the new cylinder on its posterior and lateral sides, which daily increased in hardness. At this period she was advised, by a surgeon of eminence, to have her leg amputated, but to this she would not consent. Six months after this, I saw her. She was then nearly in the situation I have just described, but the Tibia had become still more elevated, especially at its upper part, and so much so, that nearly one half of the entire substance of it, could be taken hold of by a finger and thumb. The elevation of the bone above the skin, was so great, that I attempted to divide it with a common saw, and, without wounding the skin, penetrated to some depth; after this, I endeavoured to break it, but this could not be effected. I then determined on its extraction by a pair of strong forceps, as these could readily be applied without any previous removal of the new bone or integument. The bone appeared to be strongly confined; by using, however, some force, I could perceive, that it was loose; and, on extracting forcibly, and yet slowly, (fearing least a large blood vessel might be lacerated) I got it out in a few minutes. It was seven inches and a half in length, and its upper part, was a portion of the entire cylinder of the Tibia. The saw had penetrated to the depth of three quarters of an inch through this part. The patient lost by this operation about four ounces of blood; and the cavity left by the extraction of the bone was extremely large. An amendment in every respect soon followed, but several lesser pieces of bone were afterwards extracted. She gradually recovered her health, and the use of her limb; and in a few months laid aside her crutches. Her constitution, however, was weakly and scrophulous: There were also several other ulcers in different parts of her leg, from whence the other bones were extracted. These, at times, as well as the large wound, ulcerated, and spread wider. But, as her circumstances did not admit of a removal into the country, nearly two years elapsed, before she was completely cured. A long time, however, before a complete cure took place, she had the perfect use of her leg; and she is now quite well, and very stout and strong.

"In this case, the whole of the Tibia was destroyed by the inflammation."

## Figures and Tables

**Figure f1:**
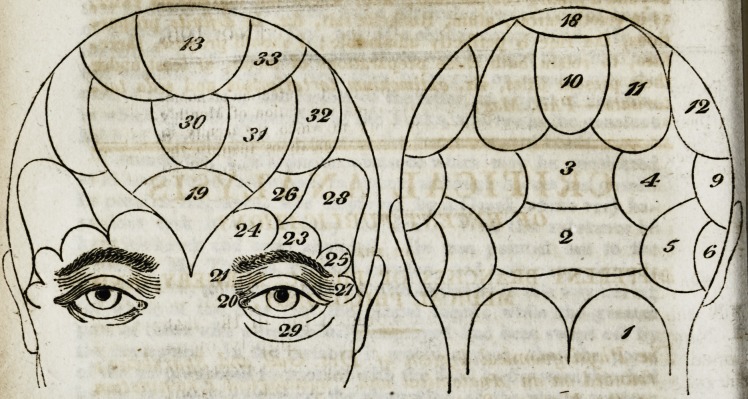


**Figure f2:**